# Supplementary material for: Near physiological spectral selectivity of cochlear optogenetics
Source: Nat Commun. 2019 Apr 29;10:1962. doi: 10.1038/s41467-019-09980-7 (PMC6488702; doi:10.1038/s41467-019-09980-7)
Supplement: Supplementary file 1 — Supplementary Information [file 41467_2019_9980_MOESM1_ESM.pdf]

Supplementary Information

Near physiological spectral selectivity of cochlear optogenetics

Alexander Dieter[1,2], Carlos J. Duque-Afonso[1,2,3], Vladan Rankovic[1,4,5], Marcus Jeschke[1,4,6,*], Tobias Moser[1,2,3,4,*]

[1]Institute for Auditory Neuroscience and InnerEarLab, University Medical Center Göttingen, 37075 Göttingen, Germany

[2]Göttingen Graduate School for Neurosciences and Molecular Biosciences, University of Göttingen, 37075 Göttingen

[3]Auditory Neuroscience Group, Max Planck Institute for Experimental Medicine, 37075 Göttingen, Germany

[4]Auditory Neuroscience and Optogenetics Laboratory, German Primate Center, 37077 Göttingen

[5]Restorative Cochlear Genomics Group, Auditory Neuroscience and Optogenetics Laboratory, German Primate Center, 37077 Göttingen

[6]Cognitive Hearing in Primates Group, Auditory Neuroscience and Optogenetics Laboratory, German Primate Center, 37077 Göttingen

* These authors jointly supervised this work

Correspondence: tmoser@gwdg.de

Supplementary Figures

Supplementary Figure 1: Linear fit of tonotopic slopes

Supplementary Figure 2: Auditory thresholds of ICC multi-units

Supplementary Figure 3: Response window of artificial SGN stimulation

Supplementary Figure 4: Intracochlear optical stimulation of non-injected gerbils

Supplementary Figure 5: Thresholds for artificial SGN stimulation

Supplementary Figure 6: Multi-peak STCs

Supplementary Figure 7: Tonotopy of artificial SGN stimulation

Supplementary Figure 8: Verifying fiber positions by Monte-Carlo ray tracing

Supplementary Figure 9: Spatial spread of excitation

Supplementary Figure 10: Effect of fiber angle on spread of excitation

Supplementary Figure 11: Spectral spread x dB above threshold

Supplementary Figure 12: Saturation of stimulus-response functions

Supplementary Figure 13: Comparison of AAV2/6 and AAV-php.b injected animals

Supplementary Figure 14: Artefact removal of electrical stimulation

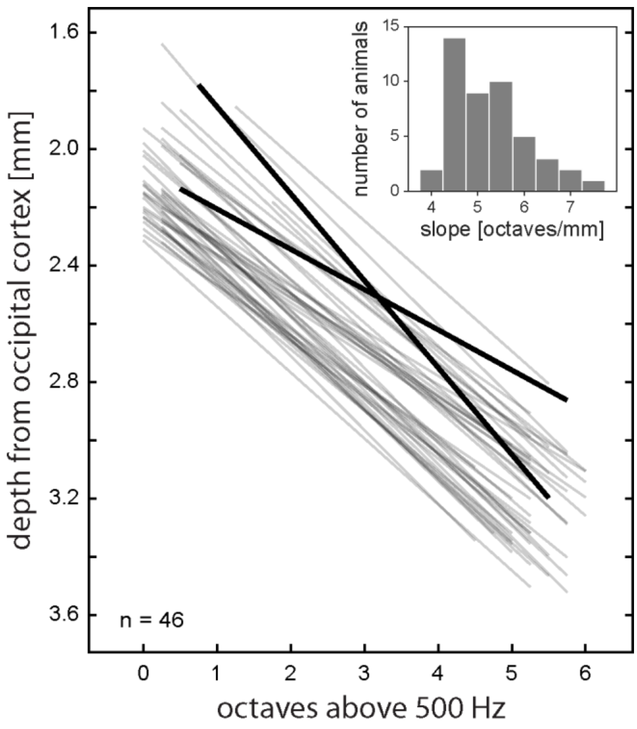

Supplementary Figure 1: Linear fit of tonotopic slopes. The tonotopic slope of each animal was calculated by a linear fit of the characteristic frequencies at given recording depths. The steepest and shallowest slopes are depicted in black lines. Inset: Distribution of tonotopic slopes in 46 gerbils contributing to this study. Source data is provided as a source data file.

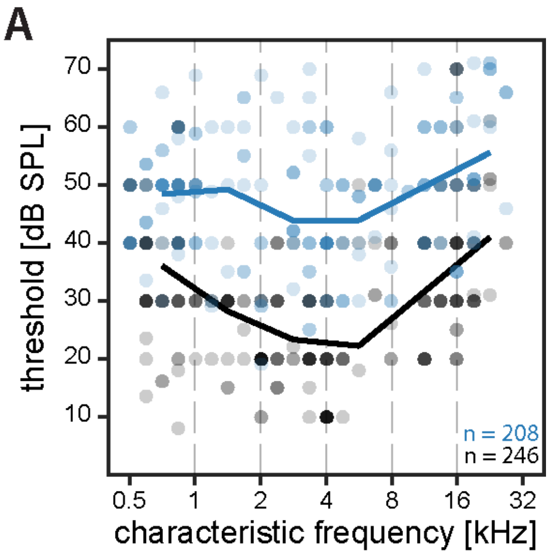

Supplementary Figure 2: Auditory thresholds of multi-units in the central nucleus of the inferior colliculus (ICC). (A) Thresholds of acoustically driven multi-units in non-injected animals without cochlear surgery (black) and in AAV-injected animals upon fiber insertion (blue). Solid lines show the mean threshold for each octave band. In animals that underwent cochlear surgery, thresholds of ICC multi-units were elevated by ~20 dB SPL on average. For each octave band, p-values are below 0.001, at least (two-sample t-test). Source data is provided as a source data file.

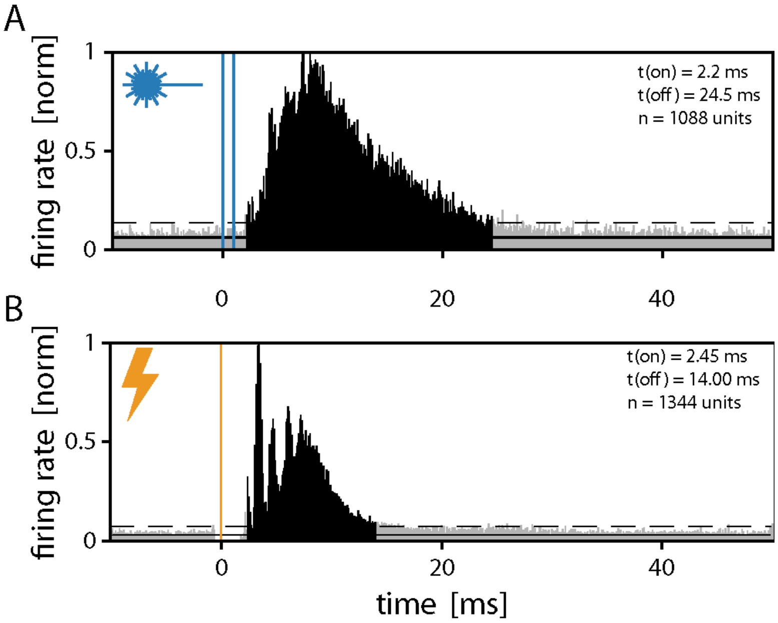

Supplementary Figure 3: Response window of artificial auditory nerve stimulation. (A) Peri-Stimulus-Time-Histogram **(**PSTH; normalized to 1) in response to optical stimulation via the round window. **(B)** PSTH (normalized to 1) in response to electrical stimulation via a cochlear implant inserted into the scala tympani via the round window. Both PSTHs are compiled across all multi-units that have been recorded upon optical or electrical stimulation. Blue and orange lines indicate stimulus on- and offset, respectively. Solid and dashed black lines indicate mean firing rate as well as the mean firing rate plus 3 standard deviations, respectively. The detected times of response on- and offsets ared epicted in the corresponding panel.

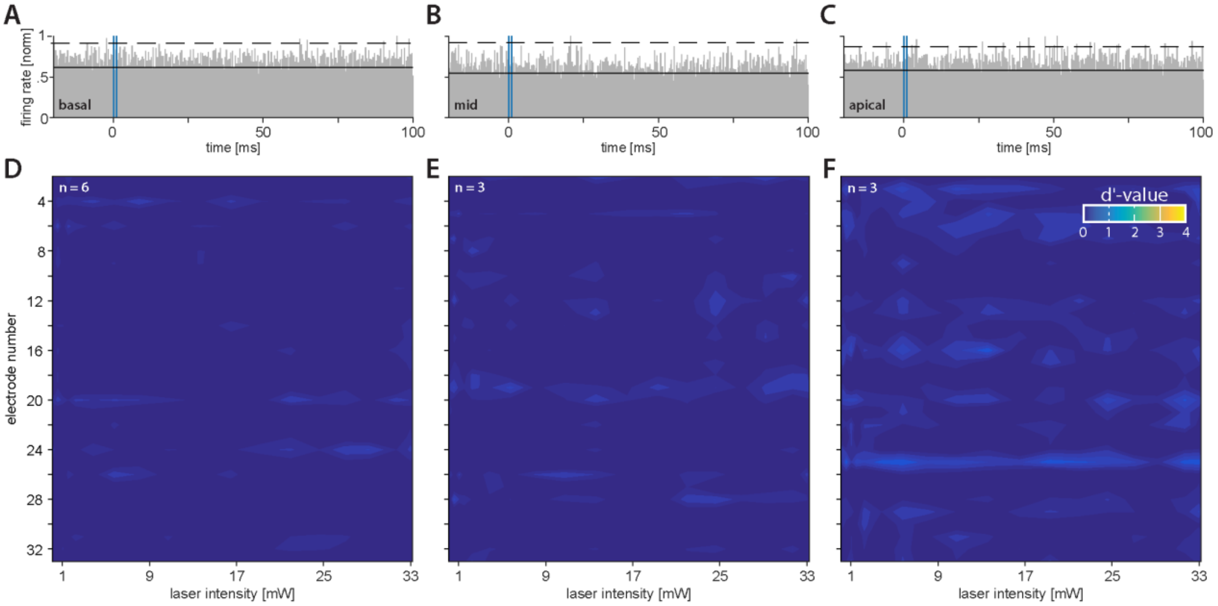

Supplementary Figure 4: Intracochlear optical stimulation of non-injected gerbils. (A-C) Peri-Stimulus-Time-Histogram (normalized) in response to optical stimulation via the round window (A; n = 6 gerbils) and cochleostomies in the middle (B; n = 3 gerbils) and apical (C; n = 3 gerbils) cochlear turn. Blue lines indicate stimulus on- and offset, respectively. Solid and dashed black lines indicate the mean firing rate as well as the mean firing rate plus 3 standard deviations, respectively. **(D-F)** Averaged spatial tuning curves (STCs) in response to basal (D) mid-turn (E) and apical (F) cochlear stimulation in non-injected animals. No responses (d' >= 1) were observed in any individual STC.

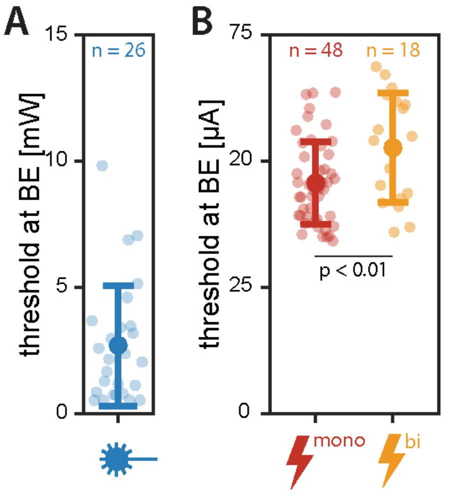

Supplementary Figure 5: Thresholds for artificial auditory nerve stimulation. (**A**) Thresholds of the best electrode (BE) for optically driven multi-units in the inferior colliculus (IC): Data is displayed as mean ± s.d.. (**B**) Thresholds of the BE for electrically driven multi-units in the IC for monopolar (red) and bipolar (orange) stimulation ($p < 0.01$, two-sample t-test). Data is displayed as mean ± s.d.. Source data is provided as a source data file.

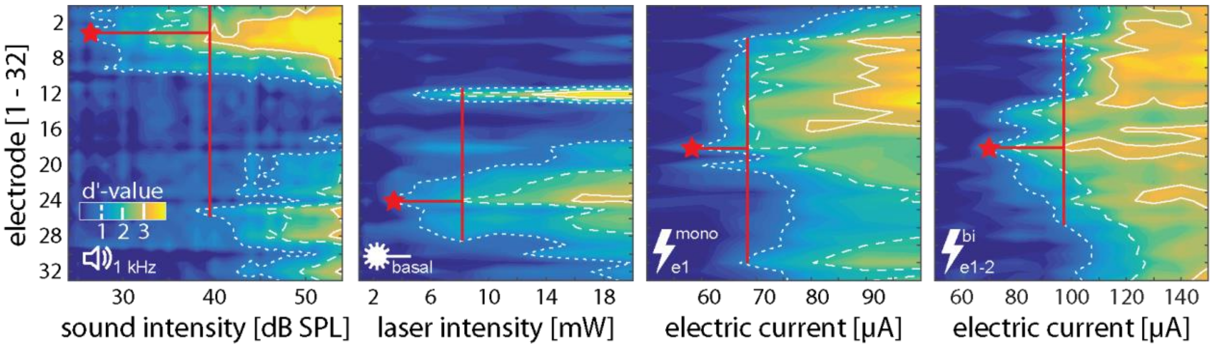

Supplementary Figure 6: Multi-peak spatial tuning curves (STCs). Few STCs with more than one peak have been observed for acoustic (left), optogenetic (center left), monopolar (center right) and bipolar (right) electrical stimulation. In these cases, the most dorsal and most ventral electrodes with significant responses have been considered as the boundaries of the STC in order not to underestimate the spread of excitation.

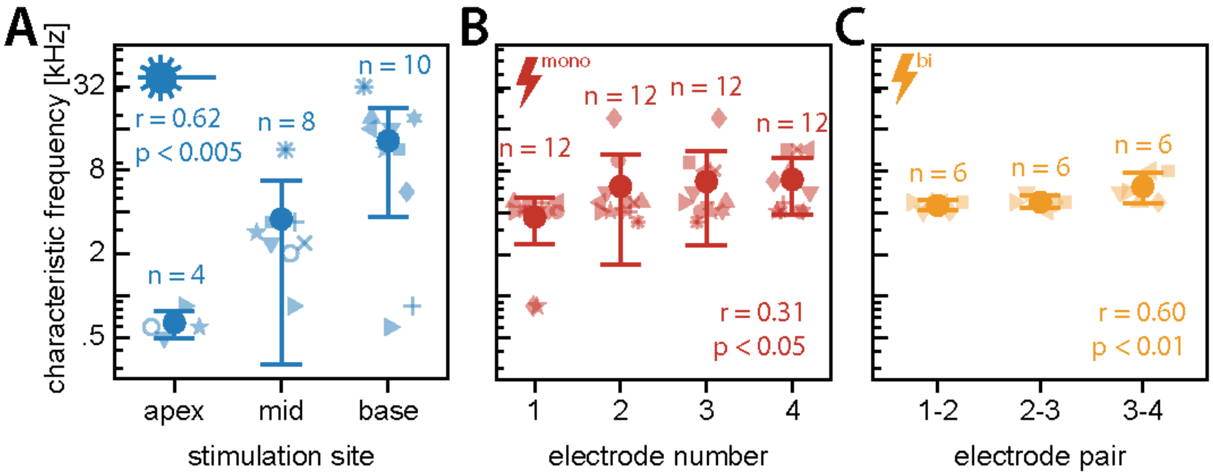

Supplementary Figure 7: Tonotopy of artificial auditory nerve stimulation. Characteristic frequencies (CFs) as a function of optical stimulation site in AAV-injected animals (**A**), stimulation electrode for monopolar electrical stimulation (**B**) and electrode pair for bipolar electrical stimulation (**C**) in non-injected animals. Data is displayed as mean and standard deviation for each stimulation site. Symbols mark different animals. Pearson's correlation coefficient *r* and the corresponding *p*-values were calculated. The two low-frequency units in response to optical stimulation from the cochlear base most likely were positioned at the border – or even outside – of the central nucleus of the inferior colliculus (ICC), where low-frequency units typically are found. However, the neural excitation upon basal cochlear stimulation always had a focus in the ventral inferior colliculus (as shown when plotting best electrodes (BEs) in dependence of stimulation site; see Fig. 4C). The discrepancy in number of data points for apical and basal optical stimulation when compared to Fig. 4C is explained by an increase in auditory thresholds upon cochlear surgery (see fig. S2), so that acoustically driven activity could not be evoked at all BEs and thus the CFs could not be determined. This primarily affected multi-units coding for frequencies at the edge of the audiogram, which typically have higher thresholds than the ones in the mid-frequency range. Source data is provided as a source data file.

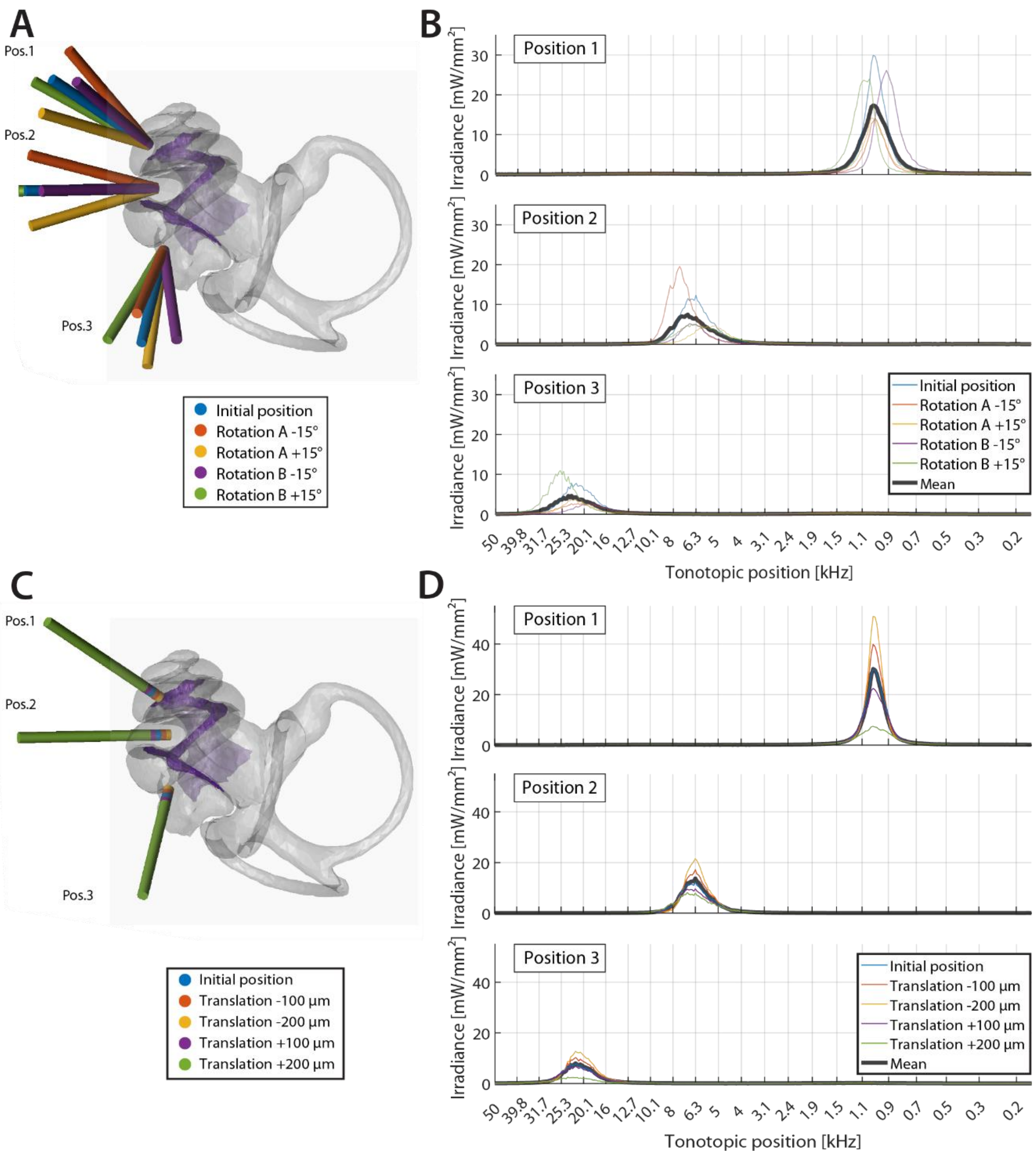

Supplementary Figure 8: Verifying fiber positions by Monte-Carlo ray tracing. 3D model reconstructed from x-ray tomography including fiber positions (Pos. 1-3), roughly corresponding to the ones used in physiological experiments. In the model, original positions as well as their respective rotation in two orthogonal planes (A and B, **panel A**) and four different coaxial translations (**C**) have been used to account for variability in fiber placement. Light grey: Bone; Grey: Scala media, vestibuli and tympani, as well as semicircular canals; Purple: Peripheral processes and Rosenthal's canal that houses the spiral ganglion neuron's somata; Fiber position and their corresponding rotations and translations, respectively, are indicated by different colors (see legend). **(B/D)** Irradiance profiles obtained from 300 query points located along the tonotopic axis, defined in the centerline of the Rosenthal's canal, upon Monte-Carlo ray tracing from the three different fiber positions using a source radiant flux of 2.67 mW. Peak irradiances of the mean traces indicate that fibers from these different positions were stimulating areas around 1.01, 6.9 and 22.89 kHz, according to the rotation model in panel B, and 1.02, 6.34 and 21.81 according to the translational model in panel D. Source data of panel B and D is provided as a source data file.

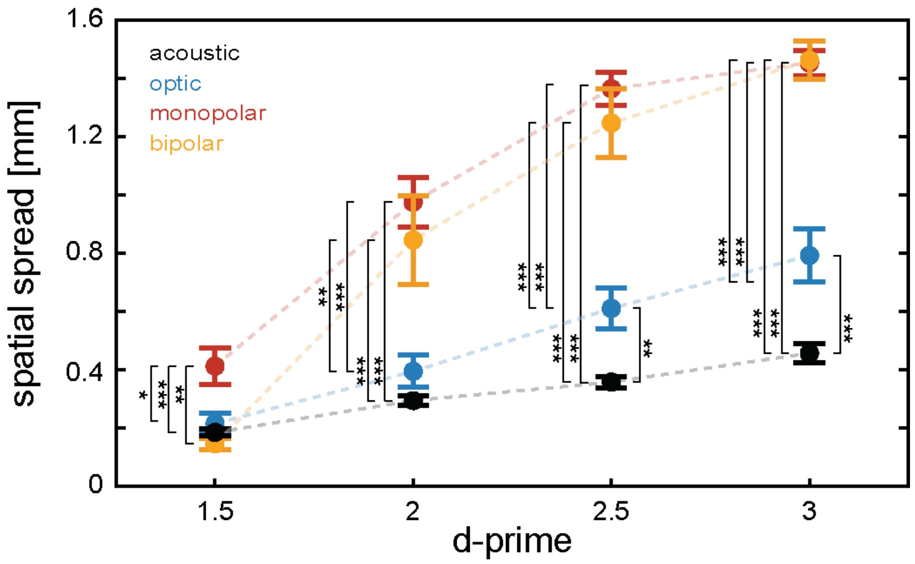

Supplementary Figure 9: Spatial spread of excitation. Mean and s.e.m. for the spatial spread of excitation upon acoustic, optogenetic, monopolar and bipolar electric stimulation. Stars indicate statistical significance (one star: $p < 0.05$, two stars: $p < 0.01$, three stars: $p < 0.001$), according to a repeated-measures Anova and post-hoc pairwise comparison tests. Only significant differences have been indicated. Source data is provided as a source data file.

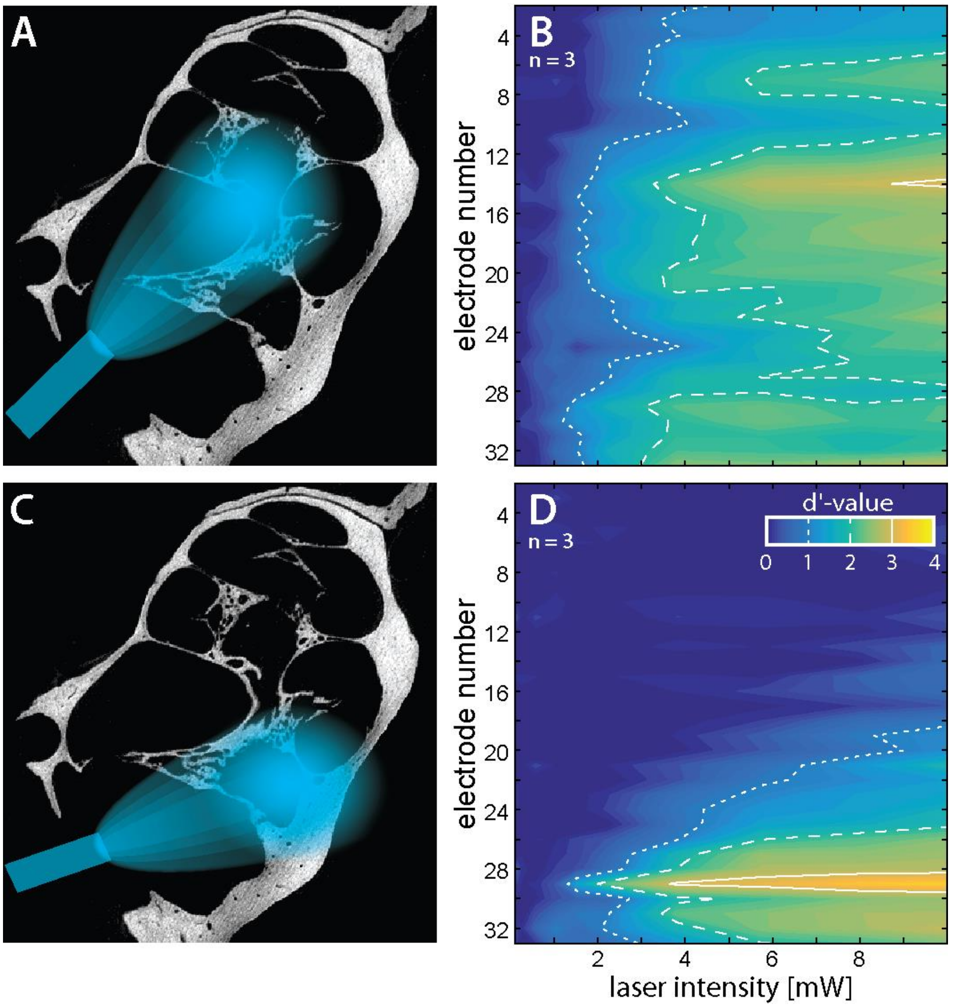

Supplementary Figure 10: Effect of fiber angle on spread of excitation. (A) Scheme of fiber placement and **(B)** resulting spread of excitation when the fiber aperture points towards the cochlear apex. **(C)** Positioning of the fiber aperture towards the cochlear base lead to more restricted spread of excitation **(D)**.

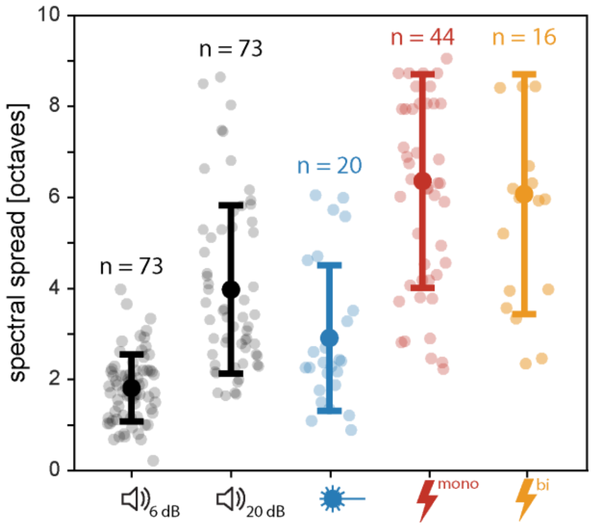

Supplementary Figure 11: Spectral spread x dB above threshold. Spectral spread in octaves, measured 6 dB above threshold of the best electrode, as done in a different study by Middlebrooks and Snyder[1] as well as 20 dB above threshold for acoustic stimulation, as done by Snyder, Bierer and Middlebrooks[2]. Data is depicted as mean ± s.d.. Source data is provided as a source data file.

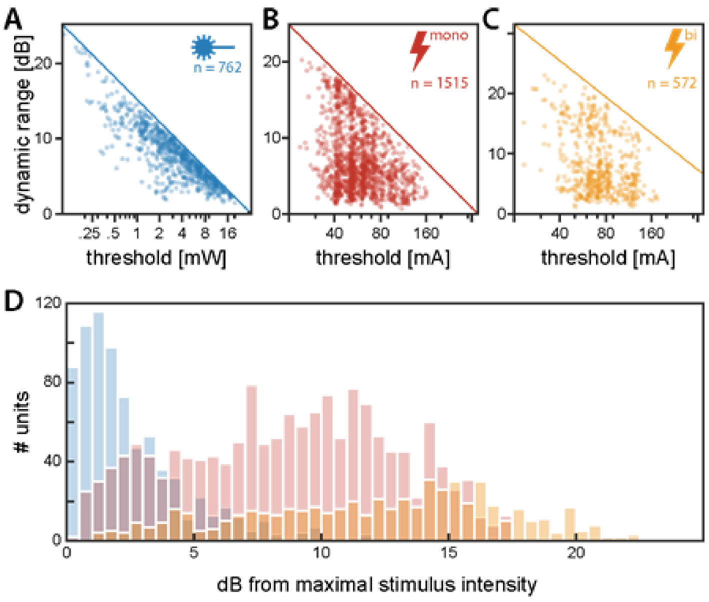

Supplementary Figure 12: Saturation of stimulus-response functions. Dynamic range (DR) of individual multi-units for optical (A), monopolar (B) and bipolar (C) electrical stimulation as a function of their respective thresholds (10% intercept). Solid lines mark the largest DR possible at the respective threshold, limited by the setup's highest stimulus intensity. While optically driven units with higher thresholds (> 2 mW) seem to be mainly limited by hardware restrictions, this was not the case for electrically driven units. (D) Difference of saturated responses (90% intercept) and the highest achievable DR (in dB). While optically driven multi-units tend to approach the highest achievable DR, electrically driven units typically were saturated at lower intensities. Source data is provided as a source data file.

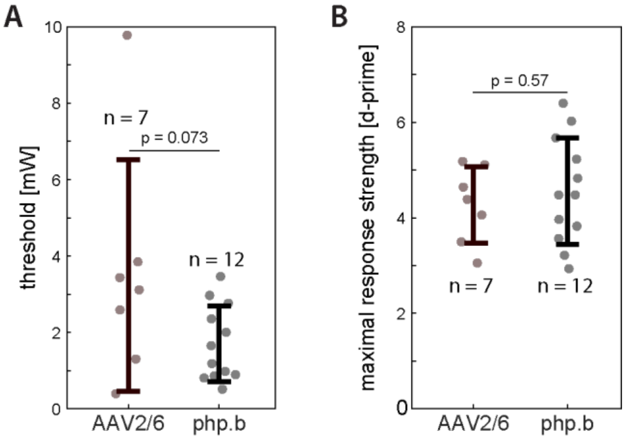

Supplementary Figure 13: Comparison of AAV2/6 and AAV-php.b injected animals. (A) Thresholds (d´ = 1) of individual animals at the best electrode(two-sample t-test; n = 7 (AAV2/6)/n = 12 (php.b)(. **(B)** Strongest response that could be evoked in each animal (two-sample t-test;n = 7 (AAV2/6)/n = 12 (php.b)). Data is depicted as mean ± s.d.. Source data is provided as a source data file.

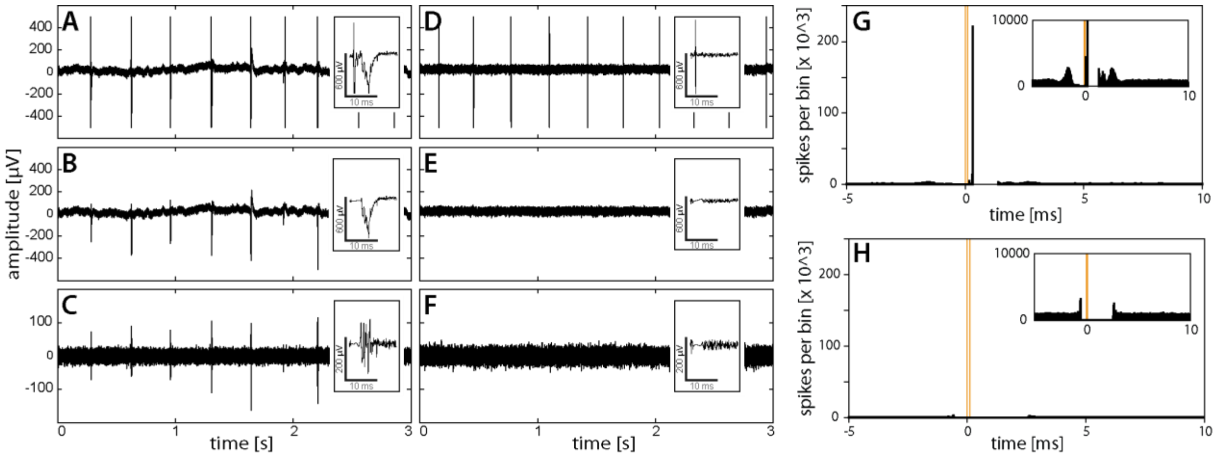

Supplementary Figure 14: Artefact removal of electrical stimulation. (A) “raw” data trace (hardware-filtered from 0.1-9 kHz) including the electrical artefacts before linear interpolation. **(B)** The same data trace after linear interpolation of a 3 ms window around the stimulation artefact. **(C)** 0.6-6kHz band-pass filtered data of the trace shown in (A) and (B) in order to extract multi-unit activity. **(D-E)** Data display as in (A-C), but here measured post-mortem in a sacrificed animal. No waveforms were observed after artefact removal. **(G-H)** Peri-Stimulus-Time-Histograms of detected waveforms upon electrical stimulation without clipping of the artefact (G) and with clipping of the artefact (H). Spike counts per bin are summed across all animals and stimulation intensities. Orange lines indicate stimulus on- and offset. Insets show a zoom-in of the y-axis.

References:

1. Middlebrooks, J. C. & Snyder, R. L. Auditory prosthesis with a penetrating nerve array. *J. Assoc. Res. Otolaryngol. JARO* **8**, 258–279 (2007).
2. Snyder, R. L., Bierer, J. A. & Middlebrooks, J. C. Topographic spread of inferior colliculus activation in response to acoustic and intracochlear electric stimulation. *J. Assoc. Res. Otolaryngol. JARO* **5**, 305–322 (2004).
