# Supplementary material for: Near physiological spectral selectivity of cochlear optogenetics
Source: Nat Commun. 2019 Apr 29;10:1962. doi: 10.1038/s41467-019-09980-7 (PMC6488702; doi:10.1038/s41467-019-09980-7)
Supplement: Supplementary file 3 — Reporting Summary [file 41467_2019_9980_MOESM3_ESM.pdf]

nature research

Corresponding author(s): NCOMMS-18-37107B

Reporting Summary

Nature Research wishes to improve the reproducibility of the work that we publish. This form provides structure for consistency and transparency in reporting. For further information on Nature Research policies, see Authors & Referees and the Editorial Policy Checklist.

Statistical parameters

When statistical analyses are reported, confirm that the following items are present in the relevant location (e.g. figure legend, table legend, main text, or Methods section).

n/a	Confirmed	
☐	☒	The exact sample size (n) for each experimental group/condition, given as a discrete number and unit of measurement
☐	☒	An indication of whether measurements were taken from distinct samples or whether the same sample was measured repeatedly
☐	☒	The statistical test(s) used AND whether they are one- or two-sided<br>*Only common tests should be described solely by name; describe more complex techniques in the Methods section.*
☐	☒	A description of all covariates tested
☐	☒	A description of any assumptions or corrections, such as tests of normality and adjustment for multiple comparisons
☐	☒	A full description of the statistics including central tendency (e.g. means) or other basic estimates (e.g. regression coefficient) AND variation (e.g. standard deviation) or associated estimates of uncertainty (e.g. confidence intervals)
☐	☒	For null hypothesis testing, the test statistic (e.g. F, t, r) with confidence intervals, effect sizes, degrees of freedom and P value noted<br>*Give P values as exact values whenever suitable.*
☒	☐	For Bayesian analysis, information on the choice of priors and Markov chain Monte Carlo settings
☒	☐	For hierarchical and complex designs, identification of the appropriate level for tests and full reporting of outcomes
☐	☒	Estimates of effect sizes (e.g. Cohen's d, Pearson's r), indicating how they were calculated
☐	☒	Clearly defined error bars<br>*State explicitly what error bars represent (e.g. SD, SE, CI)*

Our web collection on statistics for biologists may be useful.

Software and code

Policy information about availability of computer code

Data collection	Acoustic, electric and optogenetic stimulation was performed using with custom-written Matlab-Scripts (2007) and the Cheetah Software by Neuralynx (Acquisition of electrophysiological recordings). Monte-Carlo ray trace modelling was performed using the TracePro® Standard 7.8.1 Software by Lambda Research Corporation.
Data analysis	Data analysis was performed using custom-written Matlab scripts (version 2007 and 2016).

For manuscripts utilizing custom algorithms or software that are central to the research but not yet described in published literature, software must be made available to editors/reviewers upon request. We strongly encourage code deposition in a community repository (e.g. GitHub). See the Nature Research guidelines for submitting code & software for further information.

Data

Policy information about availability of data

All manuscripts must include a data availability statement. This statement should provide the following information, where applicable:

- Accession codes, unique identifiers, or web links for publicly available datasets
- A list of figures that have associated raw data
- A description of any restrictions on data availability

Data and analysis code is available from the corresponding authors (AD, MJ, TM) upon reasonable request.

Field-specific reporting

Please select the best fit for your research. If you are not sure, read the appropriate sections before making your selection.

☒ Life sciences ☐ Behavioural & social sciences ☐ Ecological, evolutionary & environmental sciences

For a reference copy of the document with all sections, see nature.com/authors/policies/ReportingSummary-flat.pdf

Life sciences study design

All studies must disclose on these points even when the disclosure is negative.

Sample size	No sample-size calculations were performed. Sample size was determined to be adequate based on the magnitude and consistency of measurable differences between groups.
Data exclusions	In 3 out of 29 optically stimulated animals, in which the cochleostomy resulted in bleeding, have been excluded from theanalysis. In these animals, optophysiological responses have been compromised by surgical complications and are thus not comparable to the remaining data set. This statement is found in the paragraph "Stimulation" in the materials/methods-section
Replication	Results were replicated in different animals within the experimental groups. No animals except the 3 before mentioned have been excluded (due to surgical complications).
Randomization	No randomization was used.
Blinding	No blinding was performed.

Reporting for specific materials, systems and methods

Materials & experimental systems

n/a	Involved in the study	
☒	☐	Unique biological materials
☒	☐	Antibodies
☒	☐	Eukaryotic cell lines
☒	☐	Palaeontology
☐	☒	Animals and other organisms
☒	☐	Human research participants

Methods

n/a	Involved in the study	
☒	☐	ChIP-seq
☒	☐	Flow cytometry
☒	☐	MRI-based neuroimaging

Animals and other organisms

Policy information about studies involving animals; ARRIVE guidelines recommended for reporting animal research

Laboratory animals	Mongolian gerbils (Meriones unguiculatus) of both sexes above the age of 8 weeks have been used for this study.
Wild animals	No wild animals have been used in this study.
Field-collected samples	No field-collected samples have been used in this study.
